# Effects of workplace stress, perceived stress, and burnout on collegiate coach mental health outcomes

**DOI:** 10.3389/fspor.2023.974267

**Published:** 2023-04-25

**Authors:** Simon A. Wright, Lauren F. Walker, Eric E. Hall

**Affiliations:** Department of Exercise Science, Elon University, Elon, NC, United States

**Keywords:** mental health, burnout, coach, stress, well-being

## Abstract

**Introduction:**

Given the continuously changing job demands of coaches, coach burnout continues to be an important area of study. Coaching literature highlights the role occupational stressors play in the development and management of burnout. However, research highlights the potential need for the field to differentiate feelings of burnout from those of other sub-clinical mental health indicators (e.g., anxiety, depression). This study sought to examine the relationship between workplace stress, perceived stress, coach burnout, coach well-being, and sub-clinical health issues (anxiety, stress, depression).

**Methods:**

One hundred forty-four NCAA collegiate coaches completed online questionnaires measuring the proposed variables. Structural equation modeling was used to test the proposed hypothesis that burnout would serve as a partial mediator between workplace and perceived stress and mental health indicators (e.g., depression, anxiety, stress, and well-being).

**Results:**

Workplace stress and perceived stress were positively associated with both burnout subscales. Additionally, perceived stress alone exhibited a positive association with depression, anxiety, and stress and a negative association with well-being. While there was a positive significant relationship between disengagement and depression in the model and a negative significant relationship between disengagement and well-being, most relationships between the two burnout subscales and mental health outcomes were negligible.

**Discussion:**

It can be concluded that while workplace and perceived life stressors may impact feelings of burnout and mental health indicators directly, burnout does not appear to exhibit a strong effect on perceptions of mental health and well-being. In line with other research studies, it may be worth considering whether burnout should be considered another type of clinical mental health issue instead of as a direct contributor to coach mental health.

## 1. Introduction

Burnout and experiences of mental health and well-being in sport, are two of the most salient issues within modern sport culture. While much attention has been paid to the athlete's experience of burnout in sport (1–4), substantially less knowledge exists about the experience of burnout in athletic coaches. This dearth of literature is concerning due to the importance of the coach role in developing life skills and high performance in athletes at various levels of sport. Additionally, research has expanded in regard to understanding the athlete's mental health challenges and experience at various levels of sport in recent years, but a similar examination of coach mental health is lacking (4, 5). A coach's experience of burnout and mental well-being (6, 7) may impact the quality of the relationship with their athletes. In seeking to understand coach burnout and mental health, researchers are undecided on whether burnout may serve as a mediator to other sub-clinical mental health indicators (e.g., depression, anxiety, well-being) or whether it should be treated as a mental health outcome in its own right (4). The purpose of this study was to propose and test an exploratory conceptual model of the relationship between workplace stress, perceived stress, coach burnout, and coach well-being and sub-clinical mental health issues (anxiety, stress, depression). Due to this study proposing and examining a conceptual model, it is important to understand what the previous literature highlights regarding the potential directional relationship between these variables in a sport coaching population.

### 1.1. Relationship between workplace stress and perceived stress on coach burnout

Burnout has often been described as a process, rather than an event, where a coach feels diminishing motivation and engagement in a previously enjoyable space (i.e., sport), and it is characterized by feelings of emotional exhaustion, depersonalization, and a reduced sense of accomplishment (8, 9). While some research alludes to a potential difference in the experience of burnout in coaching populations across gender (2, 10, 11), due to age (10), and due to coaching level (12, 13), the idiosyncratic nature of the coaching context makes it hard to examine the exact individual impact of these factors. However, what is undisputed in the literature is the role stressors, real and perceived, play on feelings of burnout (4. 9, 14, 15).

Coaches are thought to experience three unique categories of stressors within their occupation: organizational (e.g., competition preparations, sport status), performance (e.g., conflict with athletes, coaching responsibilities) and personal stressors (e.g., irregular working hours, missed family time (3, 4, 10, 16). While all three types of stressors are important to consider in long-term coach well-being and burnout, the type of stressor may be less important than the accumulation of job-related stress over time (15). Nikolaos (15) highlighted that greater accumulated feelings of job-related stress were connected to higher levels of all three indicators of burnout (emotional exhaustion, depersonalization, reduced accomplishment). Similarly, Raedeke (13) examined the feelings of coaches longitudinally, highlighting that burnout worsened as the season continued. Raedeke posited this may occur due to the increased time demands and stressors that accompany post-season play and the lack of rest that coaches get during an active season. Collectively, this research reflects that the more stressors a coach faces in their context (organizational and demands of the season), the more susceptible they may be to burnout over time.

While coaches at any level may experience stressors, coaches at the collegiate level may experience a higher number of stressors than average due to the prolonged job demands (17). Not only is there an increased likelihood of burnout development in professions where the job role is based on interpersonal relationships (18, 19), but coaches at this level must maintain elite level performance (e.g., longer playing seasons, high pressure stakes, media attention) which may tax more physical and mental resources (13). Hjalm et al. (12) examined burnout in elite soccer coaches in Sweden and found that 71% percent of women's team coaches and 23% of men's team coaches experienced moderate to high feelings of emotional exhaustion. Similarly, Malinauskas et al. (20) also highlighted a significant link between perceptions of stress and burnout among collegiate coaches. Due to these findings in the literature, a likely predictor of coach burnout is the accumulated actual and perceived stress of coaches, with coaches at a higher level (e.g., professional and college) potentially showing a stronger relationship due to the greater number of stressors their context may cause (12, 13).

### 1.2. Relationship between perceived stress and coach mental health

Given that coach mental health and well-being is multifaceted, perceived life stressors may impact not just feelings of burnout, but feelings of positive well-being, anxiety, and depression as well. While research examining the mental health impact of various performance climates has gained traction with athletes, similar examinations have been lacking within coaching populations (4, 5). Kim et al. (21) examined the prevalence of depressive feelings within an elite sport coaching population and found that perceived life stressors were a source of moderate feelings of depression. Similarly, Kaegelaers et al. (5) highlighted that 55% of elite coaches reported at least one common mental disorder (e.g., anxiety, depression).

Olusoga et al. (3) found that a failure of coaches to cope with stressors may lead to increased negative cognitions, anger/frustration, emotional fatigue, and feelings of depression. However, Smith et al. (11), found that 80% of the coaches in their sample were not aware of work-related resources to help them cope with stressors or mental health issues. Only half of the coaches in their sample felt like their boss would take expressions of mental health seriously and there was a high degree of variability in how well this would be received within their employment space. Coaches often feel they cannot share issues with mental health due to perceptions of shame, the pressurized nature of their role, and the need to appear in control (3, 11, 22). In summary, this research reflects that coping mechanisms and resources may be meaningful to reduce potential issues with mental health in coaches, but that coaches often feel a lack of personal and organizational coping resources to deal with their stressors.

Indeed, Hinogosa-Alcalde et al. (23) highlighted that the high emotional demand elite coaches face was associated with lower mental-health scores and higher feelings of burnout. Given one element of burnout is emotional exhaustion, Hinogosa-Alcalde et al.'s (23) findings may allude to elements of burnout predicting or worsening mental health outcomes in athletic coaches.

### 1.3. Relationship between coach burnout and coach mental health

Researchers remain undecided on whether burnout contributes to worsening coach mental health or whether it is a sub-clinical mental health issue itself (4). As noted in the previous section, Hinogosa-Alcalde et al.'s (23) findings reflected a possible connection between burnout and mental health indicators in coaches. McNeill et al. (7) echoed this connection in their narrative approach to understanding that emotional reactions that occur with burnout. They reported that burnout might make emotional management more difficult, due to the negative, harsh thoughts and constant rumination that burnt out coaches reported. Poor emotional management may then lead to continuing to be overinvolved emotionally, exacerbating feelings of stress and poor well-being.

While burnout does not currently exist as a clinical diagnosis, researchers have started to explore the relationship burnout has with other sub-clinical and clinical mental health conditions (4, 24). Bianchi et al. (24) highlight that part of the issue in distinguishing whether burnout is a clinical condition or a precursor to clinical conditions is past studies mistaking mild or moderate job stress as burnout. As such, they emphasize that burnout is unique and different from job stress. Further, Bianchi et al.'s (24) study showed that there is considerable overlap between the symptomatology of burnout and clinical depression. This may mean that burnout and depression exist within the same “pathological realm” (24). Olusoga et al. (4) challenge that part of the issue in understanding burnout's relationship with other clinical mental health variables is a measurement issue. Most burnout researchers acknowledge that burnout scales measure symptoms of burnout rather than the actual state of burnout, often with a survival bias (i.e., those who are truly burnt out might have left the profession pre-study). If the measurements reflect symptomatology that is similar to measurements of anxiety, well-being, or depression, it is likely difficult to distinguish between clinical burnout, depression, and prolonged fatigue (4, 24).

**Figure 1:**
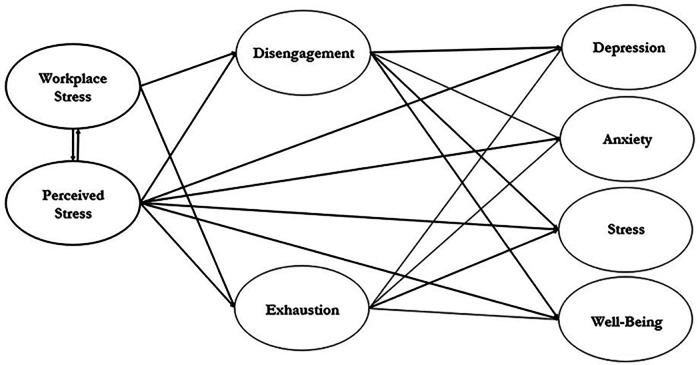
Hypothesized stress, burnout, and mental health model.

For this reason, in their scoping review of coach burnout, Olusoga et al. (4) recommend future burnout studies both measure sub-clinical mental health factors in conjunction with burnout and take occupational and life stressors into account when examining mental health and burnout. Therefore, the purpose of this study was to propose and test an exploratory conceptual model of the relationship between workplace stress, perceived stress, coach burnout, and coach well-being and sub-clinical mental health issues (anxiety, stress, depression). Given the literature reviewed above, Figure 1 reflects the proposed conceptual relationship between these variables. With burnout serving as a potential mediator between stressors and mental health outcomes in elite (collegiate) coaches.

## 2. Materials and methods

### 2.1. Participants and participant selection

To address the study purpose, an electronic survey was designed, primarily aimed at collecting quantitative data regarding stressors, burnout, and mental health; however, two open-ended questions were also included at the end of the survey. Participants' emails were taken from their institution's publicly available staff directories online. A total of 1,481 recruitment emails were sent to NCAA coaches in North Carolina. In the email, prospective participants were given information about our study and a link to the survey through Qualtrics. All participants provided informed consent and were required to provide consent before they could access the survey. Approval for the study was granted by the university's institutional review board. Emails were sent in four waves (end of March to beginning of June 2020), and three follow-up emails were sent in an attempt to generate more responses. Participants could cease participation in the study at any time and no incentive was offered for participation.

### 2.2. Instrumentation

#### 2.2.1. Demographic variables

Due to the literature highlighting the potential for contextual and demographic characteristics in coaches to contribute to experiences with organizational stressors, burnout etc. the survey instrument asked coaches to respond to a variety of demographic variables including: collegiate division coached (e.g., DI, DII), coaching level (e.g., head, assistant), race, ethnicity, gender identification, sexual orientation, sport, age, years coaching, and years at current institution.

#### 2.2.2. Mental health

The Warwick-Edinburgh Mental Well-being Scale (25) and the Depression, Anxiety and Stress Scale—21 items (26) were used to assess mental health. The WEMWBS is a 14-item scale used as a measure of positive mental health and well-being (25). This scale asks participants to rate how they have felt on a variety of statements about their feelings and thoughts over the past two weeks. An example of a statement on this scale is, “I’ve been feeling optimistic about the future.” The scale rates from 1 “None of the time” to 5 “All of the time.” The Depression, Anxiety and Stress Scale—21 items (26) contains three self-reported scales of seven items each for depression, anxiety and stress. For each item the participant rates on a scale of 0—“Did not apply to me—Never” to 3—“Applied to me very much, or most of the time—Almost Always.” Cut-off scales have been developed to define mild/moderate/severe and extremely severe scores for each subscale of the DASS-21.

#### 2.2.3. Burnout

The Oldenburg Burnout Inventory was used to measure burnout in this study (27, 28). This is a 16-item scale with two subscales, disengagement and exhaustion, which are each measured with 8 items. For each item, participants are asked to rate on a scale of 1 (strongly agree) to 4 (strongly disagree). An example disengagement item is “I find my work to be a positive challenge” while for exhaustion “When I work, I usually feel energized.”

#### 2.2.4. Perceived stress

The Perceived Stress Scale (29) is a very commonly used tool to determine an individual's perceptions of stress to a variety of situations. This is a 10-item scale where participants are asked “how often you felt or thought a certain way” with options ranging from 0 = Never to 4 = Very Often. An example item for this scale is, “In the last month, how often have you felt difficulties were piling up so high that you could not overcome them?”

#### 2.2.5. Workplace stress

Workplace stress was assessed using the Workplace Stress Survey (American Stress Institute). This is a 10-item scale on which participants rank from 1 (Strongly Disagree) to 10 (Strongly Agree) on items related to a variety of stressors related to work. An example item is “Most of the time I feel I have very little control over my life at work.”

### 2.3. Data analysis

As stated in the introduction, the primary purpose of this study was to conceptually explore a model predicting the relationship of workplace and overall perceived stress with burnout and mental health feelings in collegiate coaches. This study was approached from a structural equation model perspective due to the dearth of literature exploring the relationship of burnout to specific mental health outcomes. However, due to the abundance of literature that verifies the relationship between perceived and workplace stress with both burnout and mental health outcomes in coaches, a structural equation model seemed appropriate to explore the conceptual relationships between all variables that may impact the collegiate coach. The hypothesized latent variable model for this data appears in Figure 1. This model was used to examine whether burnout serves as a partial mediator between workplace and general perceived stress and mental health outcomes (depression, anxiety, stress, and well-being). This hypothesized model was tested with Mplus Version 8.4 statistical software (30).

## 3. Results

### 3.1. Demographics

144 NCAA collegiate coaches (*n* = 90 males; 54 females) from the state of North Carolina participated in this study. The average age of the coaching sample was 39.7 (SD = 12.3) years of age, with an average of 15.2 (SD = 11.0) years of coaching experience and a reported average tenure of 5.2 (SD = 6.5) years at their current institution. Roughly half of the sample were coaches at the NCAA Division I level (*n* = 71) and identified as the head coach (*n* = 72). The majority of the sample identified as white (*n* = 115) and heterosexual identifying (*n* = 122). Coaches of twenty different sports were represented in the sample. See Table 1 for a complete listing of the demographics for this study.

**Table 1:** Demographics of the Survey Participants.

Characteristic	*N* (144)
**Gender**	
Male	94
Female	62
Gender Non-Binary	1
**Race and Ethnicity**	
White	115
Black or African American	19
Hispanic or Latinx	3
Other reported	7
**Sexual Orientation**	
Heterosexual	122
Homosexual	12
Bisexual	5
Other	2
Not reported	3
**Coach Age**	
20–29	48
30–39	31
40–49	37
50+	32
**Years Coached**	
1–9 years	64
10–19 years	45
20–29 years	30
30 + years	17
**Years Coached at Current Institution**	
<4 years	103
5–9 years	28
10–19 years	15
20–29 years	27
30 + years	2
**Coaching Level**	
Head coach	72
Assistant/Associate coach	65
Graduate assistant/volunteer	7
**Competitive Division**	
Division I	71
Division II	44
Division III	28
Not reported	1
**Sport Coached**	
Volleyball (Indoor)	25
Basketball	19
Softball	17
Soccer	15
Track (Indoor and Outdoor)	14
Football	14
Cross Country	11
Lacrosse	10
Swimming and Diving	9
Tennis	7
Golf	6
Baseball	6
Wrestling	4
Field Hockey	4
Volleyball (Beach)	1
Rowing	1
Rifle	1
Gymnastics	1
Fencing	1
Bowling	1

### 3.2. General results

Prior to further analyses, scales and subscales used within the study survey were examined for internal consistency, *via* Cronbach alpha, with the present sample. All scales reflected acceptable reliability, and specific alpha values for each scale can be seen in Table 2.

**Table 2:** Mental health variables (M ± SD) by reported gender.

Variable	Male (*n* = 90)	Female (*n* = 54)	Total	*α*
Positive Well-Being	48.1 ± 7.2	47.8 ± 6.9	48.0 ± 7.1	0.90
Depression	2.4 ± 2.9	2.9 ± 3.2	2.6 ± 3.0	0.86
Anxiety	1.8 ± 2.5	2.1 ± 2.4	1.9 ± 2.5	0.75
Stress	4.8 ± 3.5	5.6 ± 3.2	5.1 ± 3.4	0.85
Perceived Stress	14.4 ± 6.5	17.2 ± 5.5	15.4 ± 6.3	0.87
Workplace Stress	17.2 ± 5.6	18.7 ± 5.5	17.8 ± 5.6	0.85
Disengagement	15.7 ± 4.0	15.7 ± 3.6	15.7 ± 3.8	0.79
Exhaustion	16.4 ± 3.9	17.4 ± 3.0	16.8 ± 3.6	0.82

An initial MANOVA was conducted to determine if there were differences on mental health variables by gender. There was not a significant difference in mental health variables by gender *F* (8, 135) = 1.92, *p* = 0.062. Note: The lack of significant difference present between genders, in addition to the lower sample size, led to the decision to not conduct invariance testing with the full structural equation model. See Table 2 for means and standard deviations for the variables. For further description of the sample, frequencies of the depression, anxiety, and stress subscales of the DASS-21 were used and compared to previous norms (26). From this it is seen that approximately 80% of the sample reported normal levels of depression, anxiety, and stress (See Table 3 for more description of the sample).

**Table 3:** Levels of depression, anxiety and stress (DASS-21) in collegiate coach sample.

	Depression	Anxiety	Stress
Normal	116 (80.6%)	119 (82.6%)	116 (80.6%)
Mild	15 (10.4%)	14 (9.7%)	13 (9.0%)
Moderate	10 (6.9%)	5 (3.5%)	10 (6.9%)
Severe	1 (0.7%)	4 (2.8%)	5 (3.5%)
Extremely Severe	2 (1.4%)	2 (1.4%)	0 (0.0%)

Categories were based on norms (26).

### 3.3. Structural equation model

Correlations were performed between all the variables included in the model. These found that all the variables were correlated with each other (*p* ≤ 0.001). Due to no irregularities in the parametric assumptions of the data, the maximum likelihood estimator (MLM) was used for this analysis. Per standard procedure, several fit statistics were calculated to evaluate model fit (31, 32).

**Figure 2:**
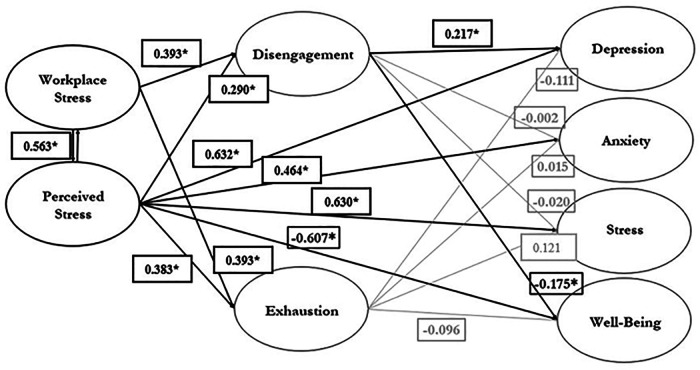
Resultant stress, burnout, and mental health model. Note: All coefficients are standardized and labeled with an * if they are significant at the *p* < 0.05 level. For ease of viewing, non-significant relationships are in light grey.

The full model results, including standardized regression path coefficients for each individual relationship, can be seen in Figure 2. Fit statistics for the hypothesized latent variable model were as follows: *χ*^2^(5) = 10.662, *p* = 0.059, CFI = 0.990, TLI = 0.948, SRMR = 0.24, and RMSEA = 0.089 (90& CI = 0.00–0.163). Per the literature (31, 32), all fit statistics reflection a reasonable to good fit, except for chi-square. Due to the extent to which chi-squared can be impacted by sample size, the fit statistics that incorporate chi-square (*χ*^2^) likely reflect a lower overall fit, due to the low sample size of the study. Note: “Good” fit for *χ*^2^ = significant; “Adequate” fit for CFI/TLI = between or equal to 0.90–0.95, “Good” fit for CFI/TLI = greater than 0.95; “Good” fit for SRMR = less than or equal to 0.08; “Reasonable” fit for RMSEA = value between or equal to 0.05–0.08.

This reasonable-good fit of the model supports the general relationships theorized in the model except for the hypothesized relationship between the exhaustion subscale of burnout and the mental health outcomes. Workplace stress (disengagement = 0.393; exhaustion = 0.393) and overall perceived stress (disengagement = 0.290; exhaustion = 0.383) were positively and significantly associated with both burnout subscales. Additionally, overall perceived stress, alone, exhibited a positive significant association with depression (0.632), anxiety (0.464), and stress (0.630), with a negative significant association with well-being (−0.607). In regard to the role of burnout as a partial mediator in the model, only one of the burnout subscales- disengagement- appeared to impact mental health outcomes. Disengagement showed a positive significant relationship with depression (0.217) and a negative significant relationship with well-being (−0.175). All other relationships between burnout subscales and mental health relationships in the model were negligible. As such, burnout did not appear to serve a strong role as a mediator between perceived/work stress and mental health outcomes.

## 4. Discussion

The conceptual model outlined at the beginning of this study resulted in a reasonable-good fit with this collegiate coaching population. This fit likely resulted from the strength of the relationship previously found between several model variables. Workplace stress and perceived stress were positively associated with both of the burnout subscales (disengagement and exhaustion), supporting the robust findings in the research that indicate organizational, performance, and personal stressors can all contribute to feelings of burnout (4, 9, 14, 15). Perceived stress emerged as the main contributor to negative mental health outcomes, as it exhibited a positive association with depression, anxiety, and stress, and a negative association with well-being. This, too, is reflective of previous literature findings (3–5).

From testing the conceptual model, it is apparent that the tentative speculation that burnout may serve to mediate the relationship between stressors and mental health outcomes was not as strong as expected. While Hinogosa-Alcalde et al. (23) suspected that higher levels of burnout, namely emotional exhaustion, might more negatively impact mental health, and McNeill et al. (7) highlighted that burnout may make emotional regulation more difficult, the results reflected negligible relationship between feelings of emotional exhaustion and mental health outcomes. Given the coaches in this sample reported depression, anxiety, stress, and well-being scores that all fell into the “normal” range, per DASS-21 scoring standards, and low levels of burnout, it is possible that the lack of the relationship appeared due to a relatively benign or manageable level of stressors or burnout in these collegiate coaches.

However, while emotional exhaustion did not reflect a strong relationship, disengagement did exhibit a significant relationship with two mental health outcomes- well-being and depression. This finding is interesting, as the disengagement items shared conceptual similarity to items from the DASS-21′s depression subscale and the Warwick-Edinburgh Well-being Scale. For example, one item from the Oldenburg disengagement subscale states: “I feel more and more engaged in my work.” One item from the DASS-21 depression subscale states: “I am unable to feel enthusiastic about anything.” Finally, a reversed sentiment on the Warwick Well-Being states: “I’ve been feeling interested in new things.” This conceptual similarity in measurement may be both reasoning for the existing significant relationships in the model and evidence to support Olusoga et al. (4) and Bianchi et al.'s (24) claims that symptoms of burnout and depression may present in similar manners and thus be hard to distinguish with current measurement tools. As such, the portion of the model linking burnout to mental health outcomes may be the reason for a less favorable fit of the overall model.

Future models might find better conceptual and statistical fit by considering burnout as a variable in line with other mental health indicators (24) instead of trying to mediate the relationship between the stress variables and mental health outcomes. Additionally, a better statistical fit may occur, due to stronger relationships between variables, if the current model were tested with elite coaches reporting high levels of burnout as an inclusion factor, similar to McNeill et al. (7).

### 4.1. Strengths and limitations

While multiple demographic variables were collected with this sample, the sample was not large enough to detect any difference in how individuals with more intersectional identities might experience job stressors, burnout, and mental health challenges. Smith et al. (11) highlight the need to attract and retain a diverse “next generation” of coaches, which means better attending to how the organizational or work climate, in particular, can create unique stressors for individuals with diverse identities.

The small sample size also impacted the fit statistics associated with the model, due to it being underpowered. While strong relationships did appear in portions of the model, and it generally showed an acceptable to good fit, further confirmatory studies should be done with larger samples to test the conceptual validity of this model. Additionally, due to the sample size, invariance testing for gender was conducted but did not produce significant results. Higher powered samples would allow for an exploration of the impact coach demographic characteristics may or may not have on the strength of relationships present in the model. While the model was underpowered, it still offers some clarification and a well-justified test of the potential relationship between perceived and workplace stress, burnout, and mental health indicators.

Finally, as has been cited by multiple researchers within the burnout and mental health literature (11), researchers should acknowledge the potential for both social desirability and survival bias to impact participant responses. In particular, survival bias may be particularly important in understanding why this sample may not have reflected high levels of burnout nor mental health issues; it may have simply been a sample that has positively coped and adapted with workplace stressors unique to the U.S. based collegiate sport system. Future research should attend to understanding the relationship in these variables over-time (longitudinally) or seek out samples of coaches that have chosen to leave their positions due to burnout or mental health issues.

## 5. Conclusion

It can be concluded that while workplace and perceived life stressors may impact feelings of burnout and mental health indicators directly, burnout does not appear to exhibit a strong mediational effect on perceptions of mental health and well-being. In line with other research studies, it may be worth considering whether burnout should be considered another type of clinical mental health issue instead of as a direct contributor to coach mental health.

## Data Availability

The raw data supporting the conclusions of this article will be made available by the authors, without undue reservation.
